# Characterization of Novel Plant Symbiosis Mutants Using a New Multiple Gene-Expression Reporter *Sinorhizobium meliloti* Strain

**DOI:** 10.3389/fpls.2018.00076

**Published:** 2018-02-07

**Authors:** Claus Lang, Lucinda S. Smith, Cara H. Haney, Sharon R. Long

**Affiliations:** Gilbert Lab, Department of Biology, Stanford University, Stanford, CA, United States

**Keywords:** root-nodule, symbiotic nitrogen fixation, bacteroid, differentiation, infection thread, fluorescent-reporter strain

## Abstract

The formation of nitrogen fixing root nodules by *Medicago truncatula* and *Sinorhizobium meliloti* requires communication between both organisms and coordinated differentiation of plant and bacterial cells. After an initial signal exchange, the bacteria invade the tissue of the growing nodule via plant-derived tubular structures, called infection threads. The bacteria are released from the infection threads into invasion-competent plant cells, where they differentiate into nitrogen-fixing bacteroids. Both organisms undergo dramatic transcriptional, metabolic and morphological changes during nodule development. To identify plant processes that are essential for the formation of nitrogen fixing nodules after nodule development has been initiated, large scale mutageneses have been conducted to discover underlying plant symbiosis genes. Such screens yield numerous uncharacterized plant lines with nitrogen fixation deficient nodules. In this study, we report construction of a *S. meliloti* strain carrying four distinct reporter constructs to reveal stages of root nodule development. The strain contains a constitutively expressed *lacZ* reporter construct; a *P_exoY_-mTFP* fusion that is expressed in infection threads but not in differentiated bacteroids; a *P_bacA_-mcherry* construct that is expressed in infection threads and during bacteroid differentiation; and a *P_nifH_-uidA* construct that is expressed during nitrogen fixation. We used this strain together with fluorescence microscopy to study nodule development over time in wild type nodules and to characterize eight plant mutants from a fast neutron bombardment screen. Based on the signal intensity and the localization patterns of the reporter genes, we grouped mutants with similar phenotypes and placed them in a developmental context.

## Introduction

Many legumes interact symbiotically with nitrogen-fixing soil bacteria, collectively called rhizobia. Rhizobium-legume symbioses involve the formation of root nodules, specialized plant organs, where bacteria receive plant-derived dicarboxylic acids in exchange for reduced nitrogen sources (Lodwig and Poole, 2003; Long, 2015). Root nodule formation is initiated when plants sense a bacterial lipochitooligosaccharide, Nod factor, which is produced by compatible rhizobia in the presence of certain flavonoids in root exudates. Detection of Nod factor by specific Nod factor receptors leads to the activation of a signaling cascade (refer to Oldroyd, 2013 for a detailed review). The activation of this signaling cascade causes modified growth of root hairs around associated bacteria, accompanied by dedifferentiation of root pericycle, endodermis and cortical cells to form the nodule primordium (Xiao et al., 2014). Rhizobia invade the plant tissue via infection threads which first form in root hairs but ultimately penetrate deep into the plant tissue (Gage, 2004). Certain legumes such as *Pisum, Trifolium, Vicia*, and the model plant *Medicago truncatula* produce indeterminate nodules with a persistent meristem at the nodule tip. In *M. truncatula* nodules, plant host cells and also bacteria within symbiosomes undergo a differentiation process characterized by transcriptional and metabolic differentiation, genome endoreduplication and morphological changes (Prell and Poole, 2006; Gibson et al., 2008; Kereszt et al., 2011; Kondorosi et al., 2013; Udvardi and Poole, 2013; Roux et al., 2014; Lang and Long, 2015). Continuous division of plant meristem cells, infection, and differentiation results in a spatial zonation of developmental stages within the nodule organ along a distal to proximal axis. Based on plant cell morphology, five zones can be distinguished in *Medicago* nodules: the meristematic zone I; infection zone II; interzone II–III; nitrogen fixation zone III; and senescence zone IV (Truchet and Dazzo, 1982). The zones are characterized by specific bacterial forms. The meristematic zone is devoid of bacteria. In the infection zone, most bacteria are present in infection threads and morphologically resemble free-living bacteria. In the interzone II-III bacteria have been released into symbiosomes and differentiate into elongated Y-shaped bacteroids, which start to fix nitrogen in the nitrogen fixation zone, and senescing bacteroids are found in the senescence zone (Vasse et al., 1990).

*Sinorhizobium meliloti* the natural symbiotic partner of *Medicago sativa* has been widely used as a model organism to study root nodule formation with *M. sativa* and *M. truncatula*. Rhizobia such as *S. meliloti* face different environmental conditions in different root nodule zones and at different developmental stages. The production of exopolysaccharides such as succinoglycan or galactoglucan was found to be critical for infection thread formation (Glazebrook and Walker, 1989; Gonzalez et al., 1996; Cheng and Walker, 1998; Pellock et al., 2000). Exopolysaccharides act as signal molecules that are recognized by a specific plant receptor in the epidermis to regulate nodule development (Kawaharada et al., 2015). In addition, exopolysaccharides can protect bacteria against oxidative stress (Lehman and Long, 2013) and they may suppress MAMP-induced plant defense responses (Aslam et al., 2008).

The *bacA* gene is prominently involved in bacteroid differentiation in interzone II–III (Kereszt et al., 2011; Kondorosi et al., 2013). *BacA* mutants fail to differentiate into bacteroids and senesce soon after their release from infection threads (Glazebrook et al., 1993). BacA protects bacteria from plant-derived antimicrobial peptides and is involved in the incorporation of very long-chain fatty acids into Lipid A (Karunakaran et al., 2010; Haag et al., 2011, 2013).

In the interzone II–III, conditions change from oxic to microoxic. This is an essential prerequisite for nitrogen fixation, because the nitrogenase enzyme is highly oxygen sensitive. In fact, genes encoding the nitrogen fixation apparatus are only expressed under microoxic conditions (Soupène et al., 1995; Bobik et al., 2006). Microoxic conditions are established by increased respiration and the expression of nodule-specific heme-proteins, the leghemoglobins, which give nodules a characteristic pinkish hue. Binding of O_2_ by leghemoglobin is essential for decreasing the abundance of free oxygen while also facilitating a high flux of oxygen to the bacteroid compartment in the nitrogen fixation zone (Ott et al., 2005).

After the bacteria are released from infection threads into symbiosomes the bacteria are exposed to new conditions, including a multitude of plant-produced nodule-specific peptides (Alunni and Gourion, 2016; Pan and Wang, 2017; Ribeiro et al., 2017). The secretion of these peptides from the plant cytoplasm into the symbiosome is essential for bacteroid differentiation. *M. truncatula* encodes more than 450 nodule-specific cysteine-rich (NCR) and glycine-rich peptides (NGR) (Kevei et al., 2002; Kereszt et al., 2011; Haag et al., 2013). Few of these proteins have been characterized at present. Some peptides have antimicrobial properties, induce bacteroid differentiation, and control bacterial infection (Van de Velde et al., 2010; Haag et al., 2011; Tiricz et al., 2013; Farkas et al., 2014), and there may be other so-far cryptic roles.

Genetic analysis has helped established the roles for plant proteins. Several plant mutants were identified that are deficient in nitrogen fixation (*dnf*) but are able to form nodules (Kuppusamy et al., 2004; Veereshlingam et al., 2004; Starker et al., 2006; Domonkos et al., 2013; Horváth et al., 2015). Characterization and mapping of these *dnf* mutations can provide information about the processes that control root nodule development. For instance, the *dnf1* mutation was mapped to a signal peptidase subunit, which is required for NCR secretion (Van de Velde et al., 2010; Wang et al., 2010). The *dnf2* mutation was mapped to a protein with a signal peptide and a phosphatidylinositol-specific phospholipase CX domain (Bourcy et al., 2012). The *dnf4* and *dnf7* mutations were mapped to the NCR peptides NCR211 and NCR169, respectively (Horváth et al., 2015; Kim et al., 2015). Study of host-microbe strain specificity has revealed a role for NCR peptides in allowing the plant to discriminate among bacterial symbionts (Pan and Wang, 2017).

Large scale Tnt1, fast neutron bombardment and ethanemethylsulfonate mutagenesis screens have yielded several hundred *M. truncatula* symbiosis mutants (Penmetsa and Cook, 2000; Wang et al., 2006; Tadege et al., 2008; Pislariu et al., 2012; Veerappan et al., 2016), most of which have not yet been characterized in detail. Initial characterization of plant nodule mutants may include staining of cellular components or bacterial cells with fluorescent or conventional dyes (Dudley et al., 1987; Haynes et al., 2004) or the use of bacterial reporter strains expressing β-galactosidase or β-glucuronidase fusions (Leong et al., 1985; Oke and Long, 1999; Starker et al., 2006). Green and red fluorescent reporter proteins such as GFP and DsRed have been used successfully to follow infection thread development (Gage et al., 1996; Gage, 2002) and to study emerging root nodules (Pistorio et al., 2002). Use of fluorescent proteins in older, fully developed nodules was found to be problematic due to diminished fluorescence in the nitrogen fixation zone (Stuurman et al., 2000), though uniform labeling was found for enhanced GFP (eGFP)-expressing *S. meliloti* in all zones of *Medicago sativa* nodules (Pini et al., 2013).

Here, we report the construction and application of a *S. meliloti* reporter strain that expresses three developmentally timed reporter genes, each at a different symbiotic stage, plus a constitutive reporter. In contrast to previous studies that used one constitutively expressed fluorophore or indiscriminate staining of all nodule bacteria, our strain can reveal the localization of different bacterial subpopulations in a nodule. We used this strain to analyze different stages of wild type *M. truncatula* nodule development, and to characterize plant mutants with impaired nodule development.

## Materials and Methods

### Plant and Bacterial Cultivation

All bacterial cultures were grown in modified Luria-Bertani medium (Meade et al., 1982). Streptomycin (500 μg/ml), neomycin (100 μg/ml), tetracycline (5 μg/ml), and spectinomcyin (50 μg/ml) were added to *S. meliloti* cultures if appropriate. For *E. coli* cultures kanamycin (25 μg/ml) was used instead of neomycin and chloramphenicol was added at 50 μg/ml if appropriate.

*Medicago truncatula* [Gaertn.] A17 and mutant seeds were lightly scratched with sandpaper and sterilized in undiluted commercial sodium hypochlorite bleach for 5 min. After rinsing with sterile water, seeds were imbibed for 8 h at room temperature and 48 h at 4°C. Seeds were germinated overnight in inverted petri dishes in the dark and planted into pre-sterilized vermiculite in cone-tainer tubes (Stuewe & Sons, Tangent). Plants were watered every third day with 5 ml 0.1× BNM. Five day old seedlings were inoculated with 5 ml of a suspension (OD_600_ = 0.1) of *S. meliloti* in 0.5×Buffered Nodulation Medium (BNM) (Ehrhardt et al., 1992).

### Construction of a Multireporter *S. meliloti* Strain

The *Sinorhizobium meliloti* strain CL150 was used as a wild type and expression host throughout this study. CL150 is a *Sinorhizobium meliloti* 1021 derivative, corrected for point mutations in *pstC* and *ecfR1* (Schlüter et al., 2013).

To generate reporter constructs, the genes encoding monomeric teal fluorescent protein gene, *mTFP* (Ai et al., 2006), and the *E. coli* β-glucuronidase, *uidA*, were PCR amplified from the plasmids pNCS-mTFP1 and pVO155, respectively (**Supplementary Table S1**). Hetero-stagger cloning was used to clone the reporter genes (Felfoldi et al., 1997) into pCAP77, which carries a *rhaS* fragment and can integrate into the *rhaS* locus in the *S. meliloti* genome (Pinedo and Gage, 2009). Basically, two PCRs with primer pairs of slightly different length (**Supplementary Table S2**) were carried out. The PCR products were gel-purified, mixed, melted and reannealed. Annealing of strands from the different PCRs resulted in the formation of sticky ends. The annealed PCR products were cloned into the KpnI site of pCAP77 generating pCL141 (*uidA*) and pCL146 (*mTFP*). The *nifH* and *exoY* promoters were amplified from CL150 genomic DNA using two sets of primers for each promoter; hetero-stagger cloning was used to integrate these promoters into the KpnI site upstream of the reporter genes in pCL141 or pCL146, resulting in pCL169 (*P_nifH_-uidA*) and pCL181 (*P_exoY_-mTFP*). Plasmid pCL169 was transferred into *S. meliloti* CL150 by conjugation and integrated into the *rhaS* locus to generate strain CL227 (Pinedo and Gage, 2009). Since genomic insertion strains of *P_exoY_-mtfp* only displayed weak fluorescence, *P_exoY_-mtfp* was amplified with primers CL348/CL351 and cloned into the EcoRV site of multicopy-plasmid pMB393, generating pLS1. To generate a *P_bacA_-mCherry* fusion, *mCherry* was amplified from pCH179 with primers CL527/CL528 and *P_bacA_* was amplified from genomic DNA using primers CL523/CL525. The two PCR products were spliced together by overlap extension PCR (Horton et al., 1989) and the resulting *P_bacA_-mCherry* PCR product was cloned into the SmaI site of pLS1 to generate pCL301. This plasmid was conjugated into CL150 to generate CL296 and into LS121, which is a CL227 derivative containing the *hemA::lacZ* plasmid pXLGD4 (Leong et al., 1985), to generate CL304. Strain descriptions are listed in **Supplementary Table S3**. Plasmids pCL301 and pXLGD4 are compatible as they have a pBBR (Kovach et al., 1995) and a pRK290 origin (Ditta et al., 1985), respectively.

### Preparation, Staining, and Microscopy of Root Nodule Sections

Root nodules were harvested by cutting the root 1 mm above and below a nodule. The three most developed nodules of ten plants were placed in cryomolds so that each cryomold contained ten nodules from different plants. The nodules were embedded with OCT (Sakura Tissue-Tek) and frozen by floating the cryomold in a polypropylene lid of a pipette-tip box on liquid nitrogen. Frozen nodules were stored at -80°C. Using a Cryotome (Microm HM550, Thermo Fisher Scientific), 25 μm sections were prepared at -20°C. The sections were transferred to adhesive coated microscopic slides (CFSA CS-1/2X, Instrumedics) using the CryoJane Tape-Transfer System (Instrumedics). The sections were incubated in phosphate buffered saline for 10 min to dissolve OCT. For the detection of fluorescent proteins, several sections from every cryoblock were mounted with mounting medium (90% glycerol, 0.01 M sodium phosphate buffer (pH 7), 0.3% w/v *n*-propyl gallate). For fluorescent GUS staining, sections were stained with 10 μM ImaGene Green C_12_FDGlcU (LifeTechnologies) diluted in 50 mM sodium phosphate buffer (pH 7) for 1 h at 37°C. The slides were washed twice before mounting. Images from whole root nodule sections were acquired with a Leica DM2500 wide field microscope equipped with a X-Cite 120LED light source and a 10× lens. Specific filter sets were used for mCHERRY, mTFP, ImaGene Green and UV autofluorescence detection. The exposure was set to either 800 ms for mTFP, mCHERRY and UV autofluorescence or to 600 ms to detect ImaGene Green. For the statistical analysis of *nifH* promoter activity in nodules from CL304 inoculated plants, root nodules were harvested at 28 dpi and bisected transversely with respect to the root axis. Five plants with an average of 10 nodules per plant were processed for each plant line. Staining for β-glucuronidase activity was performed with 5-bromo-4-chloro-3-indolyl-D-glucuronic acid (X-gluc) as described (Swanson et al., 1993). High magnification images to visualize bacteroids within the plant tissue were taken with a Leica SP5 confocal microscope and a 63× objective lens.

### Purification and Imaging of Nodule Bacteria

Nodules from *M. truncatula* A17 plants inoculated with CL304 were harvested at 17 dpi and stored at -80°C. Fifty frozen nodules from 10 plants were crushed with a microcentrifuge pestle. The tissue was resuspended in 1 ml PBS and carefully passed through two layers of Miracloth (EMD Millipore) using a syringe and filter assembly. The bacteria in the filtrate were sedimented by centrifugation at 3000 *g* for 5 min and resuspended in 200 μl PBS. GUS staining was carried out by adding ImaGene Green C_12_FDGlcU to a concentration of 10 μM and incubating at 37°C for 1 h. Stained and unstained bacteria were mounted on agarose pads (1% agarose in phosphate buffered saline (pH 7) and imaged using a wide-field microscope (Leica DM2500) and a 100× objective lens.

## Results

### Construction of a Multireporter *S. meliloti* Strain

We constructed an *S. meliloti* strain with several reporter gene fusions, each activated during different symbiotic stages, to characterize plant nodulation mutants. We selected the promoters of *exoY, bacA*, and *nifH* because these three genes were previously used to assess nodule development of plant mutants (Starker et al., 2006). The *exoY* promoter serves as a reporter for bacterial gene expression during early symbiotic stages. The *exoY* gene is the first gene of the *exoYF1Q* operon and encodes a galactosyl transferase that catalyzes the first step of succinoglycan production (Müller et al., 1993; Cheng and Walker, 1998). Succinoglycan is essential for early symbiotic stages, particularly, the initiation and elongation of infection threads (Cheng and Walker, 1998). Transcriptomic studies showed that *exoY* is expressed more highly in free-living bacteria and the nodule tip than in mature bacteroids (Barnett et al., 2004; Roux et al., 2014). The *bacA* promoter serves as a reporter for bacterial gene expression during bacteroid differentiation. The *bacA* gene is essential for the differentiation of rod-shaped bacteroids into elongated nitrogen-fixing bacteroids in galegoid root nodules (Long et al., 1988; Karunakaran et al., 2010; Haag et al., 2013). Reporter gene assays and transcriptomic studies showed that *bacA* is primarily expressed in the interzone II-III in root nodules (Glazebrook et al., 1993; Roux et al., 2014). We chose the *nifH* gene that encodes the homodimeric iron (Fe) protein (component II) of nitrogenase as a reporter for expression of the nitrogen fixation apparatus. The *nifHDK* operon includes also *nifKD*, encoding the subunits of component I, dinitrogenase [reviewed in (Fischer, 1994)]. Like other *nif* and *fix* genes, *nifH* is regulated by the FixJ/FixL two-component system, which induces expression of the nitrogen fixation apparatus via intermediate regulators FixK and NifA under microoxic conditions (Bobik et al., 2006). Therefore, *nifH* expression indicates expression of nitrogen fixation genes and reflects a low-oxygen local environment in nodules.

After testing several different reporter genes including cyan (CFP), green (GFP), red (mCherry), teal (mTFP), and flavin-binding protein derived fluorescent proteins (EcFbFp and Pp1FbFp) with an *E. coli trp* promoter (data not shown), we decided to use mTFP and mCherry to assess *exoY* and *bacA* promoter activity, respectively. As most fluorescent proteins require the presence of molecular oxygen for fluorophore formation and as non-oxygen requiring fluorescent proteins (EcFbFp and Pp1FbFp) did not yield sufficient fluorescence intensities in our hands, we used the β-glucuronidase (GUS) encoding *uidA* gene to assess *nifH* expression. Promoter fusions of *P_exoY_-mTFP* and *P_bacA_-mCherry* were cloned on a pBBR-based plasmid that replicates in *S. meliloti*. The *P_nifH_-GUS* fusion was integrated into the genome in the *rhaS* locus using the previously described pCAP77 plasmid (Pinedo and Gage, 2009). In addition to *exoY, bacA*, and *nifH* promoter fusions we also included plasmid pXLGD4, which contains a constitutively active *hemA::lacZ* fusion, as a general bacterial marker in the final multireporter *S. meliloti* strain. The *hemA::lacZ* fusion has been used in the past to visualize early symbiotic stages such as infection thread formation in root hairs (Haney and Long, 2010). While we did not use the *hemA::lacZ* fusion in this study, it would be useful in future studies characterizing early symbiosis mutants.

With a single bacterial strain able to express distinct reporters for several developmental states, it should be possible to characterize the developmental state of a plant mutant by inspection of a few nodules. We evaluated the feasibility of this approach by testing the multi-reporter strain on wild type plants and already-characterized *dnf* mutants.

### The Expression and Localization of Reporter Constructs Changes during Root Nodule Development

We inoculated wild type *M. truncatula* A17 with the multireporter strain CL304 and harvested nodules at 7, 10, 14, 17, and 21 days post infection (dpi). We prepared root nodule cryo-sections to assess reporter gene-expression by fluorescence microscopy. We found that teal (mTFP) and red (mCherry) fluorescence signals are preserved in almost all cryosections of 7, 10, 14, and 17 dpi samples but only in approximately 20% of the 21 dpi samples (data not shown). We did not pursue analysis of 21 dpi samples but focused on younger root nodules with stable fluorescence. We speculate that the environmental conditions inside the nodule change during development, resulting in conditions that do not permit fluorophore formation. It is also possible that bacterial cells may have lost the plasmid after prolonged incubation in root nodules.

**FIGURE 1:**
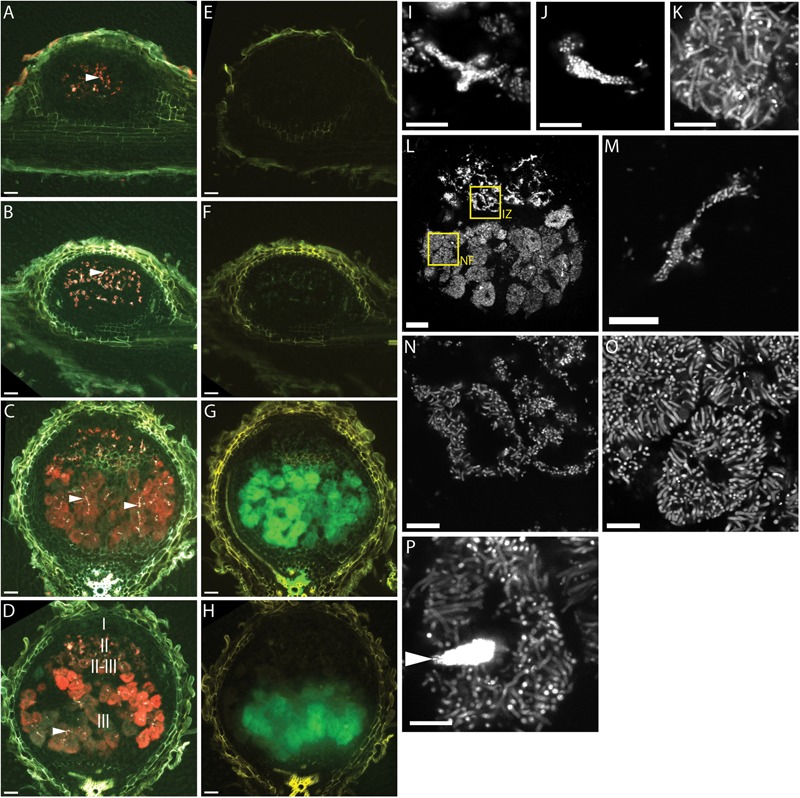
Fluorescence microscopy of wild type *Medicago truncatula* A17 root nodules at different times after inoculation with CL304. **(A–H)** Widefield micrographs of root nodules harvested 7 **(A,E)**, 10 **(B,F)**, 14 **(C,G),** and 17 **(D,H)** days after inoculation. The first column **(A–D)** shows composite images of the *P_exoY_*-*mTFP* (cyan), *P_bacA_*-*mCherry* (red), and UV-autofluorescence (yellow) signals. Due to mixing of the red and cyan signal infection threads (arrows) appear white in the images. The second column **(E–H)** shows composite images of the *P_nifH_*-*uidA* (green) and UV-autofluorescence (yellow) signals in ImaGene Green stained nodules. Images **(I–P)** are confocal images of *P_bacA_*-*mCherry* expressing CL304 bacteria in 7 **(I)**, 10 **(J)**, 14 **(K)**, or 17 **(L–P)** day old nodules. Image **(L)** is a confocal overview image of a nodule at 17 dpi. The two boxed areas indicate regions from the interzone II–III (IZ) and the nitrogen fixation zone (NF) that are magnified in images **(N)** and **(O)**, respectively. Image **(M)** is a magnification of an infection thread. Image **(P)** shows a plant cell with moderately fluorescent elongated bacteroids and an infection thread (arrow) with highly fluorescent bacteria. The different root nodule zones, I (meristem), II (infection), II–III (interzone), and III (nitrogen fixation) are indicated in image **(D)**. Scale bars: 50 μm (whole nodule images, **A–H,L**); 5 μm (confocal images, **I–K**, **M–P**).

Teal (*P_exoY_-mTFP*) fluorescence was mostly confined to infection threads in 7, 10, 14, and 17 dpi nodules (**Figures 1A–D**). This indicates that the *exoY* promoter is most active in infection threads and is turned off when bacterial cells are released from infection threads into plant cells. We detected a weak signal in bacterially invaded plant cells at 14 dpi and later time points; however, a teal-fluorescent signal of similar intensity is observed in control nodules inoculated with a non-fluorescent control strain (**Supplementary Figure S1**), thus we believe that the teal-fluorescent signal in late-stage bacterially invaded plant cells is largely due to host plant autofluorescence.

At 7 and 10 days, the red-fluorescent *P_bacA_-mCherry* is detected primarily in infection threads and the pattern matches the *P_exoY_-mTFP* signal (**Figures 1A,B**). In 14 dpi and older nodules the *P_bacA_-mCherry* signal is also detected in bacterially invaded plant cells (**Figures 1C,D**). *P_bacA_-mCherry* fluorescence was detected in invaded plant cells in the proximal part of the infection zone, the interzone II-III and in the nitrogen fixation zone. This indicates that the *bacA* promoter is active both in infection threads and in bacteroids. Considering *bacA*’s importance for bacteroid differentiation, we expected that *P_bacA_* is most active in the proximal part of the infection zone and interzone II-III. In most 14 dpi nodules we did not observe differences of the fluorescence intensity between the interzone II-III and the nitrogen fixation zone, but in some of the older, more elongated nodules *P_bacA_-mCherry* fluorescence was brightest in the interzone II-III and decreased towards the nodule base (data not shown). Using fluorescent GUS substrate ImaGene Green, we detected *P_nifH_-uidA* activity in 14 dpi and older nodules but not in 7 and 10 dpi nodules (**Figures 1E–H**). This indicates that *P_nifH_* is activated at 10–14 dpi.

The *P_bacA_-mCherry* signal intensity in bacteroids was high enough to use high magnification confocal microscopy to image single bacteroids (**Figures 1I–P**). Confocal microscopy of *mCherry*-expressing bacterial cells showed that at days 7 and 10 most bacteria are small and rod-shaped and have not yet differentiated into elongated or branched bacteroids. At days 7 and 10 a few bacteria have been released from infection threads into plant cells (**Figures 1I,J**). At days 14 (**Figure 1K**) and 17 (**Figures 1L–P**) most of the cell lumens of invaded plant cells are filled with bacteroids. In addition, undifferentiated bacteria are visible in infection threads (**Figure 1M**); undifferentiated or partially elongated cells are found in the interzone II–III (**Figure 1N**); and many fully elongated and branched bacteroids were observed in the nitrogen fixation zone (**Figure 1O**). Notably, the *P_bacA_-mCherry* signal of individual, differentiated bacteroids was only about 10–20% as bright as the signal of undifferentiated bacteria in infection threads (**Figure 1P**).

### Differences in Reporter Gene Expression and Localization in Plant Mutants with Early, Intermediate, and Late Symbiotic Defects

We calibrated the multi-reporter strain using previously characterized *dnf* mutants. We selected the mutants *dnf5* (early-arrest), *dnf2* (early-intermediate), *dnf7* (intermediate), *dnf6* (late), and *dnf3* (late) (Starker et al., 2006; Lang and Long, 2015). Since in bacteroid filled plant cells, *nifH* expression and fully developed bacteroids were visible at 14 and 17 dpi in wild type nodules, we used 14–17 day old nodules to study reporter gene expression in mutant nodules.

**FIGURE 2:**
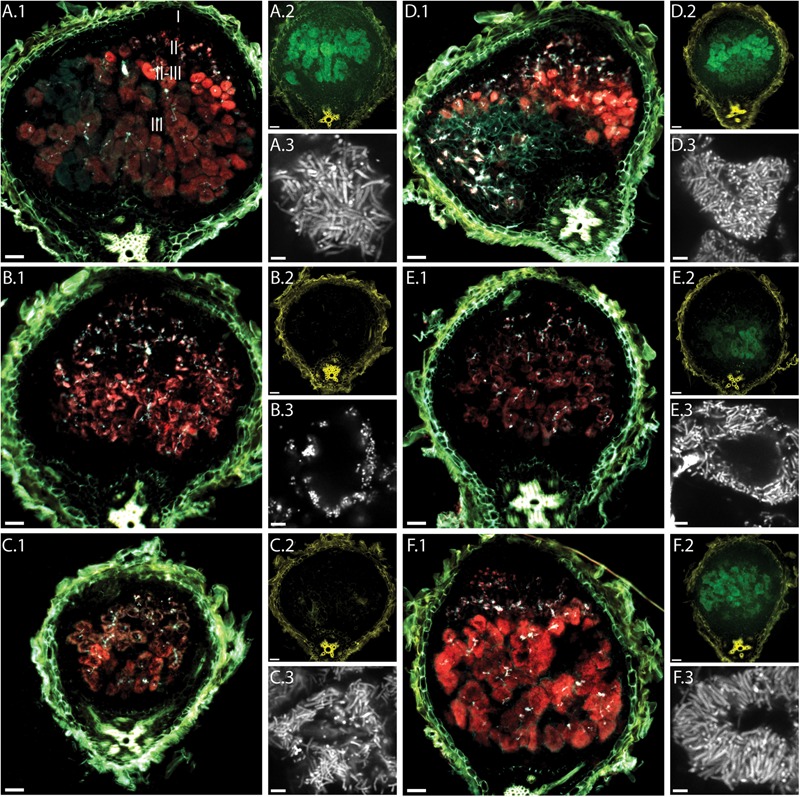
Fluorescence microscopy of root nodules from *dnf* plant mutants inoculated with CL304. Root nodules from wild type *M. truncatula* A17 **(A)** and the mutants *dnf5* **(B)**, *dnf2* **(C)**, *dnf7* **(D)**, *dnf6* **(E)**, and *dnf3* **(F)** were imaged at 14 dpi. The large images **(A.1–F.1)** are composite widefield images of the *P_exoY_*-*mTFP* (cyan), *P_bacA_*-*mCherry* (red), and UV-autofluorescence (yellow) signals. Images **(A.2–F.2)** are composite widefield images of the *P_nifH_*-*uidA* (green) and UV-autofluorescence (yellow) signals in ImaGene Green stained nodules. Images **(A.3–F.3)** are confocal images of *P_bacA_*-*mCherry* labeled bacterial cells within root nodules. The different root nodule zones, I (meristem), II (infection), II–III (interzone), and III (nitrogen fixation) are indicated in image **(A.1)**. Scale bars: 50 μm (wide field images); 5 μm (confocal images).

Analysis of the *P_exoY_-mTFP* signal revealed the presence of infection threads in nodules of all tested plant lines (**Figure 2**). This confirms that bacteria enter nodules of these *dnf* mutants. In 14 dpi nodules, the infection threads are found not only near the nodule tip but also near the nodule base. Similar localization patterns and fluorescence intensities for invading bacteria suggest that infection thread formation is not affected by any of the *dnf* mutants.

We detected red fluorescence signals in all *dnf* mutants, indicating that bacteria in all the selected *dnf* plant mutants express *bacA*. In some cases, (e.g., **Figure 2A**) we observed a region of very bright fluorescence in the first layer of bacterially invaded plant cells. This was occasionally observed in larger, more developed nodules formed by A17, Jemalong and *dnf3*, but not in the other mutants. It is possible that at 14 dpi *bacA* is highly expressed in most bacteroids since they are either in the process of differentiation or have just finished differentiation, whereas for more developed nodules *bacA* expression is turned off in the fully differentiated bacteroids. The *mCherry* expression patterns reveal differences between the *dnf* mutants in the localization of bacteroids within plant cells. In the intermediate- and late-arrest mutants, *dnf7* and *dnf3*, we observed strong *P_bacA_-mcherry* signals in the interzone and the nitrogen fixation zone. Similarly, to wild type nodules, invaded plant cells in the nitrogen fixation zone of *dnf7* and *dnf3* mutants appeared almost completely red, indicating that bacteroids completely fill out plant cells in the nitrogen fixation zone and that vacuoles become very small. In contrast, in the earlier mutants *dnf5* and *dnf2* the *P_bacA_-mCherry* signal is mostly confined to the cell periphery indicating that bacteroids are only found near the cell wall of invaded plant cells in *dnf2* and *dnf5* nodules. In *dnf6* nodules, the *P_bacA_-mcherry* signal was not as strong as in the other nodules. Similar to *dnf2* and *dnf5* nodules, the signal was localized mainly in the cell periphery in nodules from the *dnf6* mutant, which, due to a low level of nitrogenase activity, was previously classified as a late-arrest mutant (Starker et al., 2006; Lang and Long, 2015). Confocal imaging confirmed that larger parts of the invaded plant cells in *dnf2, dnf5*, and *dnf6* nodules than in nodules from other *dnf* plants were devoid of bacteroids. In addition, we observed differences in cell shape between the different mutants. The bacteroids in *dnf5* nodules were not elongated and of similar length as the undifferentiated bacteria in infection threads. The bacteroids in *dnf2* and *dnf6* were not as large as the bacteroids of the wild type plants but they were longer than the infection thread bacteria or bacteroids in *dnf5* nodules. The largest, most-elongated bacteroids were found in wild type, *dnf7* and *dnf3* nodules. Our findings suggest that bacteroid maturation and/or proliferation is affected by the mutations in *dnf5, dnf2*, and *dnf6* nodules but not in *dnf7* and *dnf3* nodules.

We used a fluorescent GUS substrate to detect *P_nifH_-uidA* activity in nodule sections. The fluorescence intensities in the different nodule sections showed that at 14 dpi the *nifH* promoter is active in wild type, *dnf3, dnf6*, and *dnf7* nodules. In contrast, we did not detect any GUS activity in *dnf2* and *dnf5* nodules indicating that the activity of the *nifH* promoter is very low or absent.

### Characterization of Potential Nodulation Mutants

**FIGURE 3:**
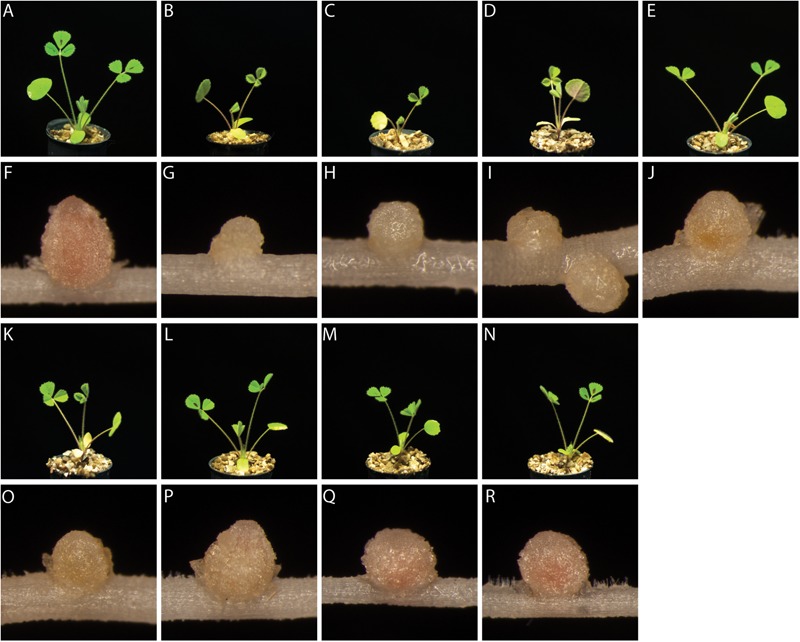
Plant and nodule morphology of FNB plant mutants. Shoots (rows 1 and 3) and root nodules (rows 2 and 4) from wild type *M. truncatula* A17 **(A,F)** and the mutants 6168 **(B,G)**, 6265 **(C,H)**, 6488 **(D,I)**, 6359 **(E,J)**, 6469 **(K,O)**, 6470 **(L,P)**, 6471 **(M,Q),** and 6701 **(N,R)** were imaged at 24 dpi.

**FIGURE 4:**
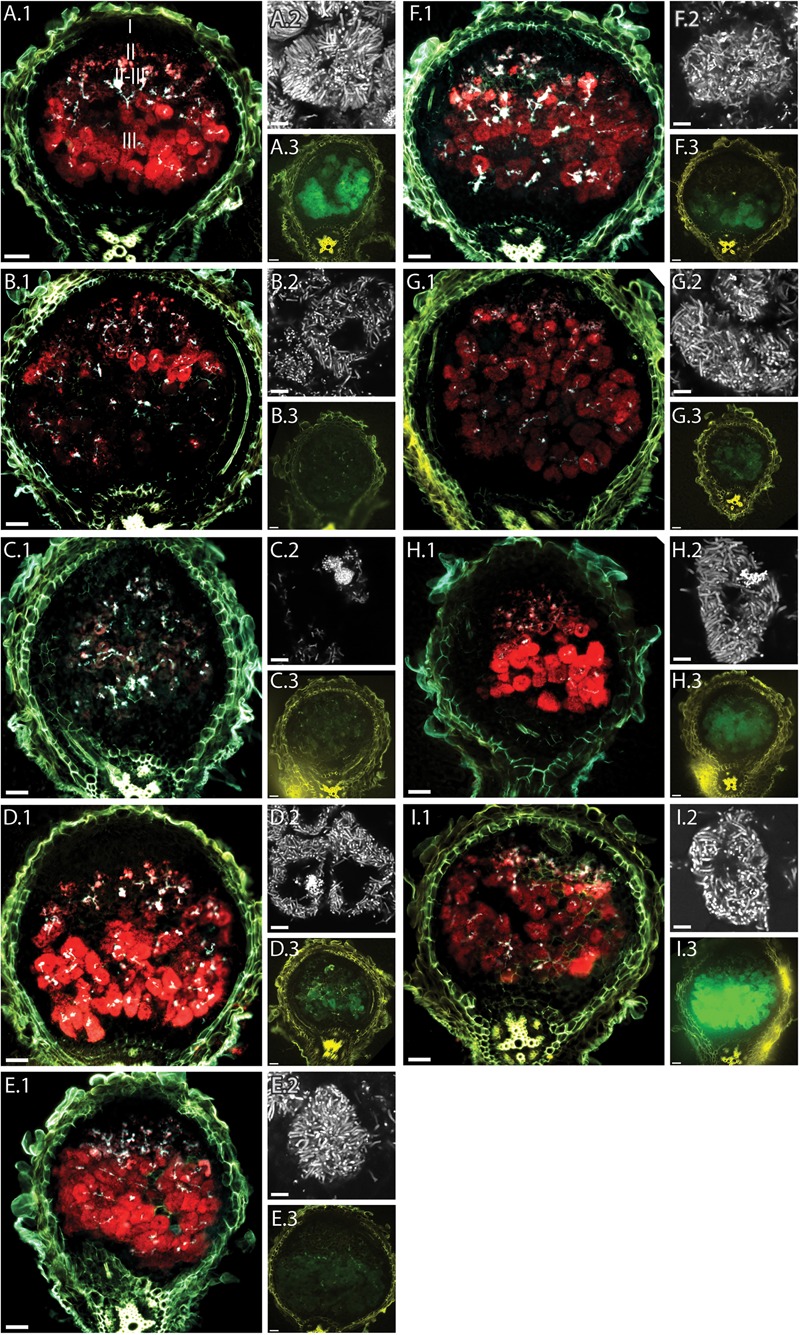
Fluorescence microscopy of root nodules from FNB plant mutants inoculated with CL304. Root nodules from wild type *M. truncatula* A17 **(A)** and the mutants 6168 **(B)**, 6265 **(C)**, 6488 **(D)**, 6359 **(E)**, 6469 **(F)**, 6470 **(G)**, 6471 **(H)**, and 6701 **(I)** were imaged at 17 dpi. The large images **(A.1–I.1)** are composite wide-field images of the *P_exoY_*-*mTFP* (cyan), *P_bacA_*-*mCherry* (red), and UV-autofluorescence (yellow) signals. Images **(A.2–I.2)** are confocal images *P_bacA_*-*mCherry* labeled bacterial cells within root nodules. Images **(A.3–I.3)** are composite widefield images of the *P_nifH_*-*uidA* (green) and UV-autofluorescence (yellow) signals in ImaGene Green stained nodules. The different root nodule zones, I (meristem), II (infection), II–III (interzone), and III (nitrogen fixation) are indicated in image **(A.1)**. Scale bars: 50 μm (wide field images); 5 μm (confocal images).

Since the reporter strain proved useful to characterize and classify nodulation mutants, we applied the CL304 reporter strain to putative *M. truncatula* symbiosis mutants that were not previously characterized. We studied eight fast neutron bombardment (FNB) mutant plant lines from the *M. truncatula* mutant collection at the Samuel Roberts Noble Foundation. The descriptions from the primary screen indicate that all eight FNB mutants have a Fix– phenotype (**Table 1**). After 4 weeks of cultivation, the shoots of three mutants (6168, 6265, 6488) were stunted in comparison to wild type plants (**Figure 3**). The leaves of these plants appeared yellowish, showing signs of chlorosis. The root nodules of these plants were small, white and spherical whereas wild type nodules were larger, pink and slightly elongated. Two mutants (6359, 6469) had normal-sized shoots but the leaves appeared light green, indicating starting chlorosis and the nodules were small, spherical, and not as pink as wild type nodules. After 4 weeks of inoculation with *S. meliloti* CL304, we did not see strong differences in terms of shoot size, leave, and nodule color between three mutants (6470, 6471, 6701) and the wild type. Fluorescence microscopy of cryosections showed that teal *P_exoY_-mTFP* fluorescent signals are present in nodules of all mutants (**Figure 4**). This indicates that the nodules from all tested mutants are invaded by bacteria. Activity of the *exoY* promoter further indicates that succinoglycan biosynthesis is activated in infection thread bacteria as in wild type nodules. The infection thread localization pattern in mutant plant lines was similar to the infection thread pattern of wild type nodules and the infection threads were evenly distributed over different nodule zones in all plant lines. This suggests that infection thread formation is not affected in any of the new FNB-mutants. By contrast, the *P_bacA_-mCherry* expression patterns differed between the mutants. In one mutant (6265) the red fluorescence signal was almost completely absent in invaded plant cells (**Figure 4**). This indicates that intracellular bacteria do not express *bacA* and do not differentiate normally. Confocal microscopy revealed that most bacteria in these nodules remain in infection threads and only very few are released into plant host cells. Some of the released bacteria were slightly elongated, suggesting that differentiation is initiated but that the bacteria die before the process is completed. This mutant therefore appears to have a distinct phenotype.

**Table 1:** Summarized results of fast-neutron-bombardment plant mutant characterization.

Plant line	Description from the Samuel Roberts Noble foundation	Plant phenotype (size, leave color)	Nodule phenotype	Whole nodule P*_nifH_-uidA* staining	*P_exoY_-mTF*P	P*_bacA_-mCherry*	*P_nifH_-uidA*	Confocal
A17		Normal size, green	Pink	++	IT over whole nodule	Bright signal, bacteria completely fill plant cells in NZ	++	Elongated bacteroids
6168	Nodulation: Fix–	Small, chlorotic	White	-	IT over whole nodule	Bright signal in IZ, very faint signal in NZ	-	Elongated bacteroids
6265	Nodulation: Fix–/green nodules	Small, chlorotic	White	-	IT over whole nodule	Faint signal in plant cell periphery	-	Most bacteria remain in infection threads, only few partially elongated bacteroids
6488	Nodulation: Fix–, Stem: reduced stem	Small, chlorotic	White	-	IT over whole nodule	Very bright signal, bacteria completely fill plant cells in NZ	+	Elongated bacteroids
6359	Nodulation: Fix–, Root: aborted primary root Stem: dwarf	Normal size, light green	Pale pink	+	IT over whole nodule	Bright signal, bacteria completely fill plant cells in NZ	+	Elongated bacteroids
6469	Nodulation: Fix–, Leaf: misshapen, no chevrons	Normal size, light green	Pale pink	+	IT over whole nodule	Bright signal, bacteria completely fill plant cells in NZ	+	Elongated bacteroids
6470	Nodulation: Fix+/–	Normal size, green	Pink	+	IT over whole nodule	Bright signal in IZ, weaker signal in NZ, bacteria completely fill plant cells in NZ	+	Elongated bacteroids
6471	Nodulation: Fix–	Normal size, green	Pink	++	IT over whole nodule	Bright signal, bacteria completely fill plant cells in NZ	++	Elongated bacteroids
6701	Nodulation: Fix–	Normal size, green	Pink	++	IT over whole nodule	Bright signal in IZ, very faint signal in NZ, bacteria completely fill plant cells in NZ	++	Elongated bacteroids

*Fix, Nitrogen fixation deficient; IT, infection thread; NZ, nitrogen fixation zone; IZ, interzone II–III*.

In the nodules from mutant 6168, the *P_bacA_-mcherry* signal is mostly confined to the infection zone and the interzone. Only a few infection pockets display red fluorescence in the nitrogen fixation zone. Magnification of the cells in the interzone (**Figures 4B.2**) shows bacteria are released into plant cells and differentiate into elongated bacteroids. The absence of intracellular red fluorescent signals in the nitrogen fixation zone suggests that bacteroids senesce prematurely in 6168 nodules.

In nodules of the remaining mutants (6488, 6359, 6469, 6470, 6471, 6701) the *P_bacA_-mcherry* signal was seen in the infection zone, interzone and nitrogen fixation zone. In all six cases the appearance of bacterially invaded plant cells resembles nodules of intermediate (*dnf4*), late (*dnf3*), or wild type nodules as the invaded plant cells appear to be almost completely filled with bacteroids. This indicates that bacteria are able to survive within plant host cells. Confocal imaging demonstrated the presence of elongated bacteroids in all six plant lines, indicating that the bacteroids differentiate and that genome endoreduplication occurs.

**FIGURE 5:**
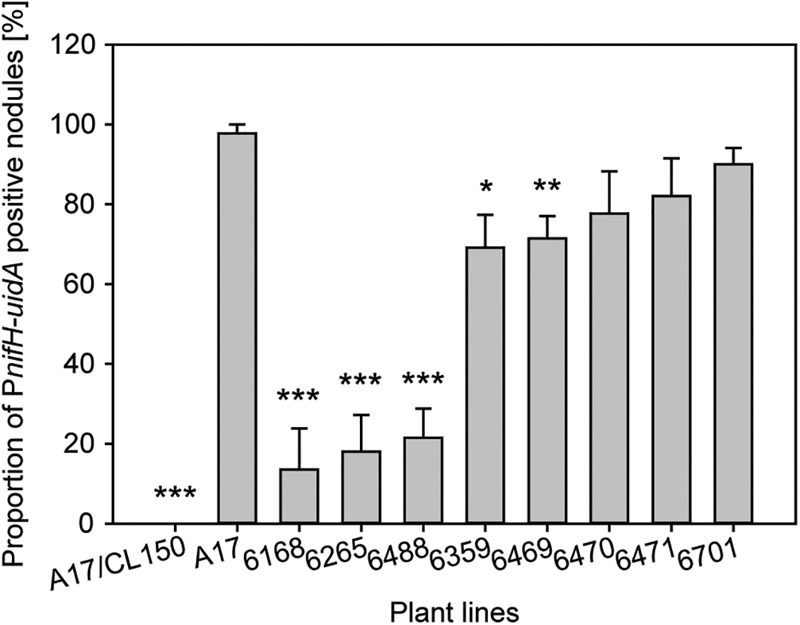
Expression of bacterial *nifH* inside FNB mutant plant nodules. Expression of *P_nifH_*-*uidA* promoter fusion inside the nodules of wild-type and FNB mutant plants was assayed at 28 dpi in five plants per plant line. Values are expressed as the percentage of positive-staining nodules in comparison to all nodules. The error bars indicate the standard error of the mean. Asterisks indicate significant differences (^∗^*p* < 0.05; ^∗∗^*p* < 0.01; ^∗∗∗^*p* < 0.001) in comparison to wild type according to two-tailed, heteroscedastic Welch’s *T*-statistics (Welch, 1947).

We used two different methods to assess expression of the *P_nifH_-uidA* reporter fusion. First, we stained nodules from several plants with the non-fluorescent GUS dye, X-gluc, and calculated the percentage of stained, *P_nifH_*-positive nodules at 28 dpi. While almost all wild type nodules displayed GUS activity the proportion of *P_nifH_* positive nodules was strongly reduced in some of the FNB plant lines (**Figure 5**). Only approximately 20% of the nodules from lines, 6168, 6265, and 6488 and approximately 70% of the nodules from lines 6359 and 6469 showed *P_nifH_* activity (**Figure 5**). The percentage of *P_nifH_*-positive nodules seemed also to be reduced in lines 6470, 6471, 6701, but the difference to wild type was not found to be statistically significant.

In addition, we employed a fluorescent GUS dye, ImaGene Green, to study the localization of the *P_nifH_* promoter activity in cryosections of younger nodules. We saw no *P_nifH_-uidA* signals in cryosections of 6168 and 6265 sections; observed faint signals in 6488, 6359, 6469, and 6470; and strong signals in 6471, 6701, and wild type nodules (**Figure 4**). Even though imaging ImaGene Green stained cryosections of 17dpi nodules (**Figure 4**) and counting the number of X-Gluc positive nodules of 28 dpi plants (**Figure 5**) are very different methods to assess nifH expression similar trends are observed. For instance, X-gluc staining and ImaGene Green staining indicate that nitrogenase expression is severely impaired in 6168 and 6265. For plant line 6488, we observed a strong reduction of *P_nifH_* positive nodules in 28 dpi plants (**Figure 5**) and low level of *P_nifH_* activity in the cryosections of 17 dpi plants. It is possible that nitrogenase expression is not sustained in 6488, that nitrogenase expression occurs in an insufficient number of nodules, or that nitrogenase expression is too weak. Considering the strong plant phenotype (**Figure 3**), our results are consistent with the conclusion that nitrogenase expression is severly impaired in 6488 nodules. X-Gluc and ImaGene Green staining experiments are consistent with a slightly impaired nitrogenase expression in 6359, 6469, and possibly 6470 nodules while there appears to be normal nitrogenase expression in 6471 and 6701 nodules. In most plant lines (6469, 6470, 6471, 6701, A17), *P_nifH_* activity was observed in the nodule center and the nodule base, where the nitrogen fixation zone is usually found in wild type nodules. In 6359, nodules the *P_nifH_-uidA* signal was occasionally seen in the root-proximal third of the nodule but not in the nodule center (**Figure 4**). This suggests that the expression of nitrogenase subunits may be delayed in 6359 nodules. A much larger sample than the 10–15 nodules tested here by the ImaGene Green assay could quantify the reproducibility and the degree of any delay for 6359 nodule maturation. Nodules from FNB mutants displayed similar fluorescence patterns if harvested at 14 (not shown) or 17 dpi. The results of all FNB mutant analyses are summarized in **Table 1**.

### Staining and Microscopy of Purified Nodule Bacteria

**FIGURE 6:**
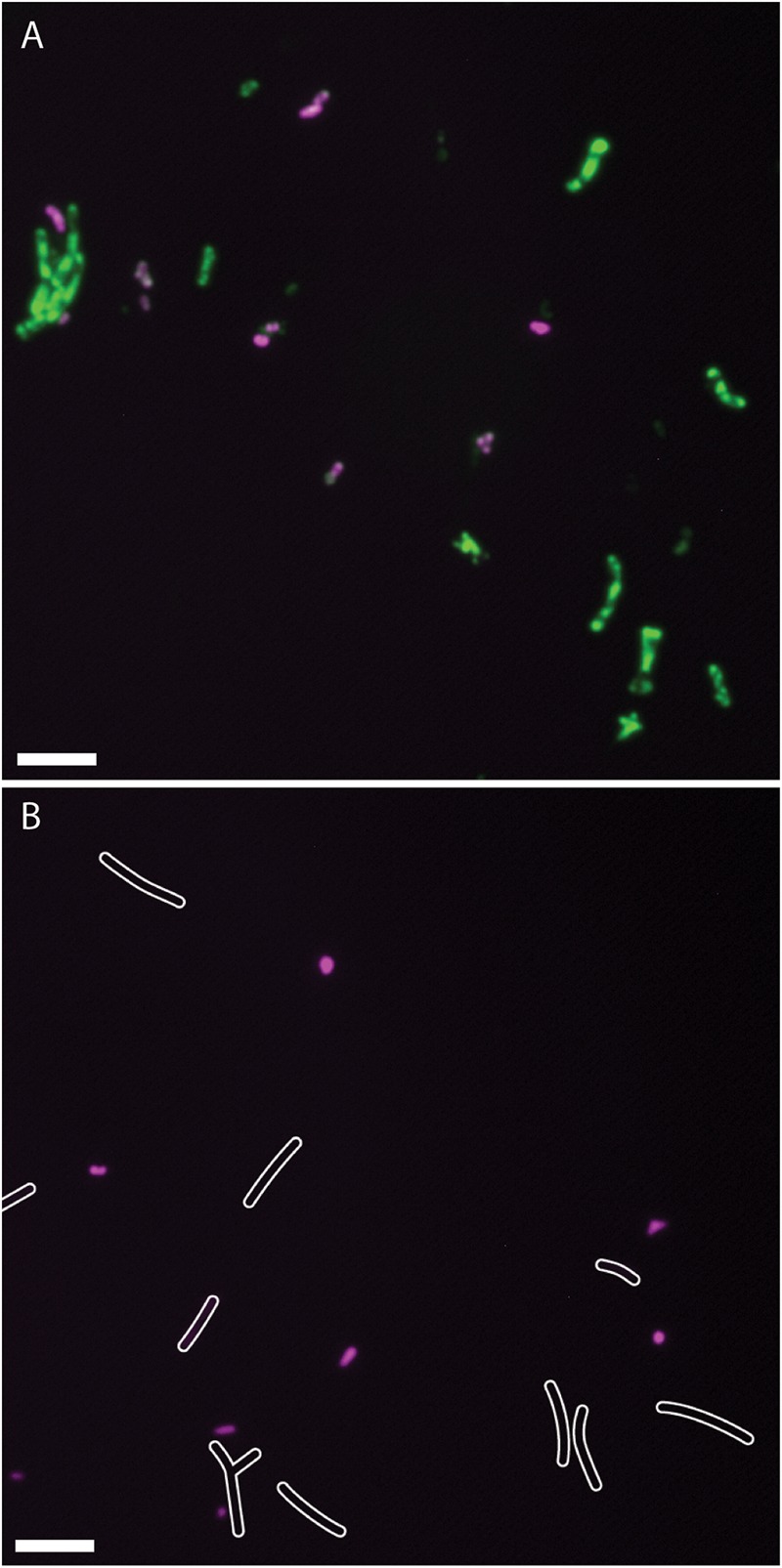
Fluorescence micrographs of *Sinorhizobium meliloti* CL304 cells purified from root nodules. Bacteria were purified from 17 dpi wild type *M. truncatula* A17 nodules and assayed for the expression of *P_bacA_*-*mCherry* (magenta) and *P_nifH_*-*uidA* (green). Bacteria were imaged either after staining for *uidA* activity with ImaGene Green **(A)** or without staining **(B)**. White cell outlines were added based on differential interference contrast imaging to indicate the position of weakly fluorescent bacteria in the unstained image. Scale bars: 5 μm.

Since the CL304 multireporter was useful to visualize different bacterial populations such as infection thread bacteria and nitrogen fixing bacteroids in the nodule, we were interested if different bacterial populations could also be distinguished by the expression of different marker proteins after extraction from root nodules. To separate bacterial cells from the plant tissue, we crushed frozen nodules and filtered the cell suspension through a Miracloth membrane. Microscopy showed that the filtrate contained bacteria of varying sizes (**Figure 6**). The teal fluorescence of our nodule bacteria preparation was very weak, and it was not possible to detect *P_exoY_-mTFP* signals reliably from single cells. We detected strong *P_bacA_-mcherry* signals in small, undifferentiated, rod-shaped bacteria but not in elongated or branched bacteroids. This was unexpected, because we detected *P_bacA_-mcherry* expression in individual, elongated bacteroids in cryosections. However, confocal microscopy showed that the signal of individual cells was weaker in differentiated bacteroids than in undifferentiated bacteria (**Figure 1P**). It is possible that the *P_bacA_-mcherry* signal of purified bacteroids was too weak to be detected by conventional wide-field fluorescence microscopy. After staining with ImaGene Green, *P_nifH_-uidA* signals were only detected in differentiated, elongated bacteroids. Therefore, the CL304 multireporter strain can be used to distinguish populations of mostly undifferentiated bacteria, that express *bacA* but not *nifH*, from elongated, mostly differentiated bacteria, that do express *nifH* but not *bacA*, based on cell fluorescence.

## Discussion

The characterization of plant and bacterial symbiosis mutants has been central to elucidating the initial symbiotic plant–bacterial signal exchange and to identifying processes that control cell differentiation between both symbiotic partners (Kereszt et al., 2011; Oldroyd, 2013). Large-scale Tnt1, FNB, and EMS mutagenesis screens yielded several hundred *M. truncatula* mutants with defects during later symbiotic stages (The Samuel Roberts Nobel foundation; http://medicago-mutant.noble.org/mutant/; (Penmetsa and Cook, 2000; Wang et al., 2006; Tadege et al., 2008). So far, few of these mutants have been characterized in detail. Here, we describe the construction of a bacterial multireporter strain for the characterization and initial classification of symbiosis mutants. In addition to a constitutively expressed *lacZ* fusion this strain contains a *P_exoY_-mTFP* fusion that fluoresces in infection threads but not in differentiated bacteroids, a *P_bacA_-mCherry* fusion that fluoresces in infection threads and in differentiated bacteroids and a *P_nifH_-uidA* fusion which indicates nitrogenase expression. The expression of different reporter genes during different developmental stages makes this strain a useful tool to study root nodule development. Previous uses of fluorescent proteins to label nodule bacteria relied largely on constitutively expressed reporter genes (Stuurman et al., 2000; Pistorio et al., 2002; D’Haeze et al., 2004; Auriac and Timmers, 2007; Pini et al., 2013). We adapted a fluorescent GUS staining protocol and developed a cryosectioning procedure that is compatible with wide-field and confocal fluorescence microscopy. The fluorescent GUS staining of a *P_nifH_-uidA* fusion can be used in conjunction with autofluorescent proteins such as mTFP and it provides a highly sensitive alternative to a previously published NifH-GFP fusion (Gavrin et al., 2014). Our procedure makes it possible to observe differences between wild type and mutant nodules in only 2 weeks old nodules. While most fluorescence microscopy studies of root nodules rely on the use of fresh nodule tissue that is processed immediately after nodule harvest (Stuurman et al., 2000; Haynes et al., 2004; Auriac and Timmers, 2007; Domonkos et al., 2013), the use of frozen tissue makes it possible to harvest and analyze root nodules on different days. Using a cryosectioning procedure it is also possible to section multiple nodules simultaneously. Therefore, the cryosectioning procedure makes it possible to cultivate, harvest and section nodules from many plants in parallel, which is essential for medium and high throughput applications.

Analysis of *dnf* and FNB plant mutants with the *P_exoY_-mTFP* fusion showed that nodules from all tested strains contained *exoY*-expressing bacteria in infection threads. Since *P_exoY_-mTFP* is expressed specifically in the infection threads, it could be useful to visualize defects in the infection thread network. Our observations here on both the previously known *dnf* and the new FNB mutants compared to wild type indicate that the mutations in all these plants act downstream of infection thread formation.

When we designed the multireporter strain we expected the *P_bacA_-mCherry* fusion specifically to label differentiating bacteroids in interzone II–III. While we observed higher *P_bacA_-mcherry* fluorescence in individual undifferentiated bacterial cells than in differentiated bacteroids by high resolution confocal microscopy, we observed similar fluorescence levels in infection thread bacteria, in differentiating bacteria of interzone II–III and in fully differentiated bacteroids of zone III using widefield fluorescence microscopy. In widefield microscopy, the intensity difference between differentiated and undifferentiated cells is not as pronounced because the focal plane is broader and signal from multiple layers of bacteroids is detected. The *P_bacA_-mCherry* fluorescence pattern revealed several differences between the mutants. In one plant line (6168) we detected very little *P_bacA_-mCherry* fluorescence indicating that bacteroids do not start to differentiate and/or senesce immediately after release from infection threads in this mutant. Other mutants such as *dnf5* and *dnf6* displayed unusual *P_bacA_-mCherry* localization patterns in invaded plant cells. Confinement of the *P_bacA_-mCherry* signal to the cell periphery suggests that the vacuoles in these mutants are increased in comparison to wild type. The strong *P_bacA_-mCherry* fluorescence signal enabled us to study the morphology of bacteria within infection threads and invaded plant cells by confocal microscopy and to distinguish mutants with apparently undifferentiated, partially elongated and fully elongated bacteroids. Overall the *P_bacA_-mCherry* fusion has proven useful to detect defects in bacterial release from infection threads, bacteroid differentiation and bacteroid survival.

A *P_nifH_-uidA* fusion serves as an indicator for nitrogenase gene expression. We observed a wide range of *P_nifH_-uidA* activities in the different mutants, from severely impaired *P_nifH_-uidA* activity in mutants with bacteroid differentiation or survival defects (*dnf5, dnf2*, 6168, and 6265 mutants) to intermediate activities (*dnf7*, 6488, 6359, 6469, 6470, and 6471) and wild type activities in lines *dnf3* and *6701.* Our results for *nifH* expression in the *dnf7* mutant differed slightly from the patterns seen by Horvath et al., as we observed *nifH* expression in the interzone and distal parts of the nitrogen fixation zone whereas Horvath et al. only observed *nifH* expression in cells in the interzone (Horváth et al., 2015). The differences may reflect changes of the *P_nifH_-uidA* localization pattern with nodule age, since we used 14 and 17 dpi nodules whereas Horvath et al used 21 dpi nodules. Since *nifH* expression is controlled by the FixL/FixJ/NifA regulatory cascade that is mainly activated by microaerobic growth conditions (Soupène et al., 1995; Bobik et al., 2006), low and intermediate *P_nifH_-uidA* activity might arise from defects in the establishment of microaerobic conditions in the nodule. For certain late symbiotic mutants, such as *dnf3* and 6701 we cannot detect any differences in *P_nifH_-uidA* activity in comparison to wild type. In these cases, processes downstream of nitrogenase expression such as nitrogenase assembly, nitrogenase stability or nutrient exchange between host and microbe may be defective.

Based on microscopic analysis with multi-reporter strain CL304, it is possible to classify nodulation mutants according to different symbiotic stages. The mutants with the earliest and most severe defects are 6265 and *dnf5*. Bacteria do not differentiate into elongated bacteroids at all in *dnf5* nodules; in 6265, only a few cells are found outside of infection threads, and these bacteria seem to senesce before bacteroid differentiation is completed. Based on these phenotypes, we place the 6265 mutations after the previously characterized IPD3, *lin* and *nip* mutants, which have defects in infection thread formation (Kuppusamy et al., 2004; Veereshlingam et al., 2004; Domonkos et al., 2013). The phenotype of the *dnf5* mutant is similar to that of the previously described *dnf1* mutant (Wang et al., 2010), which is defective in NCR peptide maturation and secretion. In *dnf1* and *dnf5* nodules, bacteria are released from infection threads into plant cells but do not show signs of differentiation. Behavior of the CL304 multireporter strain in *dnf1* nodules was indistinguishable from that in *dnf5* nodules (data not shown). This is in agreement with previous studies that found strong similarities between the *dnf5* and the *dnf1* gene expression profiles (Lang and Long, 2015). In three mutants (6168, *dnf2, dnf6*) bacteroids were only partially elongated. In 6168 mutant nodules, bacteria are released from infection threads and undergo differentiation but seem to senesce rapidly. Similar phenotypes with greatly reduced bacterial abundance in the nitrogen fixation zone were observed in *dnf8*, 5L/11S, and 13U mutants (Domonkos et al., 2013). In contrast to 6168, we found partially elongated bacteroids in all zones of *dnf2* nodules at 14 dpi which suggests that bacteroid senescence is not as rapid in *dnf2* nodules as in 6168 nodules. Premature nodule senescence has also been reported for *dnf2* nodules (Bourcy et al., 2012). In *dnf6* nodules bacteroids were also not fully elongated. However, in contrast to *dnf2* and 6168 nodules, we detected *P_nifH_-uidA* activity in *dnf6* nodules. This suggests that the *dnf6* mutation is not as severe as the *dnf2* mutation and while the *dnf6* mutation seems to affect bacteroid morphology it may not completely block root nodule development.

Mutants *dnf3, dnf7*, 6488, 6359, 6469, 6470, 6471, and 6701 supported the differentiation of fully elongated bacteroids. In comparison to wild type, *P_nifH_-uidA* activity was reduced in 6488, 6359, 6469, and 6470 mutants but not in *dnf7, dnf3*, and 6701 nodules. With the exception of *dnf7* for which we detected wild type like *P_nifH_* expression, our *P_nifH_-uidA* expression results in *dnf* strains are in good agreement with the results from Starker et al., who detected highest *P_nifH_-uidA* activities in *dnf3* and *dn6*, intermediate activity in *dnf4* and *dnf7*, low activity in *dnf2* and no activity in *dnf1* and *dnf5* nodules (Starker et al., 2006).

When placing the mutants in a developmental context, we note that the FNB mutants have not been backcrossed yet. Therefore, it is possible that a plant line carries several mutations that affect root nodule development. Since there is biological variability even between different root nodules formed by the same plant it is important to study several nodules of the same plant line. In this study, we selected the biggest nodules from 10 to 15 plants per plant line. Using our cryosectioning procedure it was possible to separate nodule harvest and analysis, and to section and stain 10–15 nodules simultaneously. However, since we deliberately selected the most developed nodules of each plant we would not be able to detect a difference to wild type if the phenotype of a mutant was leaky and only a fraction of the nodules were affected. The multireporter strain provides a tool to rapidly characterize plant mutants and should help to identify mutants that warrant further detailed biochemical and genetic characterization.

We have shown in this study that a bacterial multireporter strain with *P_exoY_-mTFP, P_bacA_-mCherry*, and a *P_nifH_-uidA* fusion can be used to characterize *M. truncatula* root nodule mutants. We chose the *P_exoY_, P_bacA_*, and *P_nifH_* promoters because these genes have important functions during different symbiotic stages. In future, other promoters could be selected based on recent transcriptomic data (Roux et al., 2014; Lang and Long, 2015) to target other bacterial nodule subpopulations. In addition to analyzing plant mutants, it was possible to detect reporter gene expression in bacteria that were isolated from root nodules. Therefore, the reporter strain could be used to quantify different bacterial nodule populations. The strain may also facilitate fluorescence activated cell sorting to collect specific bacterial populations for transcriptomic, proteomic or biochemical experiments.

## Author Contributions

LS and CL cultivated plant lines and harvested nodules. LS, CL, and CH constructed bacterial reporter strains. CL carried out cryosectioning, nodule staining, and microscopy. SL, CL, and CH conceived the study and designed experiments. SL and CL wrote the manuscript.

## Conflict of Interest Statement

The authors declare that the research was conducted in the absence of any commercial or financial relationships that could be construed as a potential conflict of interest.

## Supplementary Material

FIGURE S1 Fluorescence microscopy of *Sinorhizobium meliloti* control strains. The first row **(A–E)** shows nodules from plants inoculated with the reporter-less, wild type strain CL150. The second row **(F–J)** shows nodules inoculated with strain CL227, which contains the *P_nifH_-uidA* fusion but not the pCL301 plasmid with *P_exoY_-mTFP* and *P_bacA_-mCherry*. The third row **(K–O)** shows nodules inoculated with CL296, which contains the pCL301 plasmid but not the *P_nifH_-uidA* fusion. The fourth row **(P–T)** shows nodules from the final reporter strain CL304. The first column **(A,F,K,P)** shows the *mTFP* signal, the second column **(B,G,L,Q)** shows the *mCherry* signal, the third column **(C,H,M,R)** the UV signal, the fourth column **(D,I,N,S)** an overlay of the *mTFP, mCherry*, and UV signal, and the fifth column **(E,J,O,T)** shows an overlay of the *uidA* and UV signals. All nodules were harvested at 14 dpi nodules from *M. truncatula* A17. Scale bar: 50 μm.

TABLE S1 Plasmids used in this study.

TABLE S2 Primers used in this study. fw - forward primer; rev - reverse primer; P - 5′-phosphorylated primer.

TABLE S3 *S. meliloti* strains used in this study.
