# Corrigendum: Characterization of Novel Plant Symbiosis Mutants Using a New Multiple Gene-Expression Reporter *Sinorhizobium meliloti* Strain

**DOI:** 10.3389/fpls.2018.00848

**Published:** 2018-06-13

**Authors:** Claus Lang, Lucinda S. Smith, Cara H. Haney, Sharon R. Long

**Affiliations:** Gilbert Lab, Department of Biology, Stanford University, Stanford, CA, United States

**Keywords:** root-nodule, symbiotic nitrogen fixation, bacteroid, differentiation, infection thread, fluorescent-reporter strain

Cara H. Haney was not included as an author in the published article. The authors apologize for this error and state that this does not change the scientific conclusions of the article in any way.

New Author Contribution Statement:

LS and CL cultivated plant lines and harvested nodules. LS, CL, and CH constructed bacterial reporter strains. CL carried out cryosectioning, nodule staining, and microscopy. SL, CL, and CH conceived the study and designed experiments. SL and CL wrote the manuscript.

The original article has been updated.

## Conflict of interest statement

The authors declare that the research was conducted in the absence of any commercial or financial relationships that could be construed as a potential conflict of interest.

