# Supplementary figures and images for: Characterization of Novel Plant Symbiosis Mutants Using a New Multiple Gene-Expression Reporter Sinorhizobium meliloti Strain
Source: Front Plant Sci. 2018 Feb 7;9:76. doi: 10.3389/fpls.2018.00076 (PMC5808326; doi:10.3389/fpls.2018.00076)

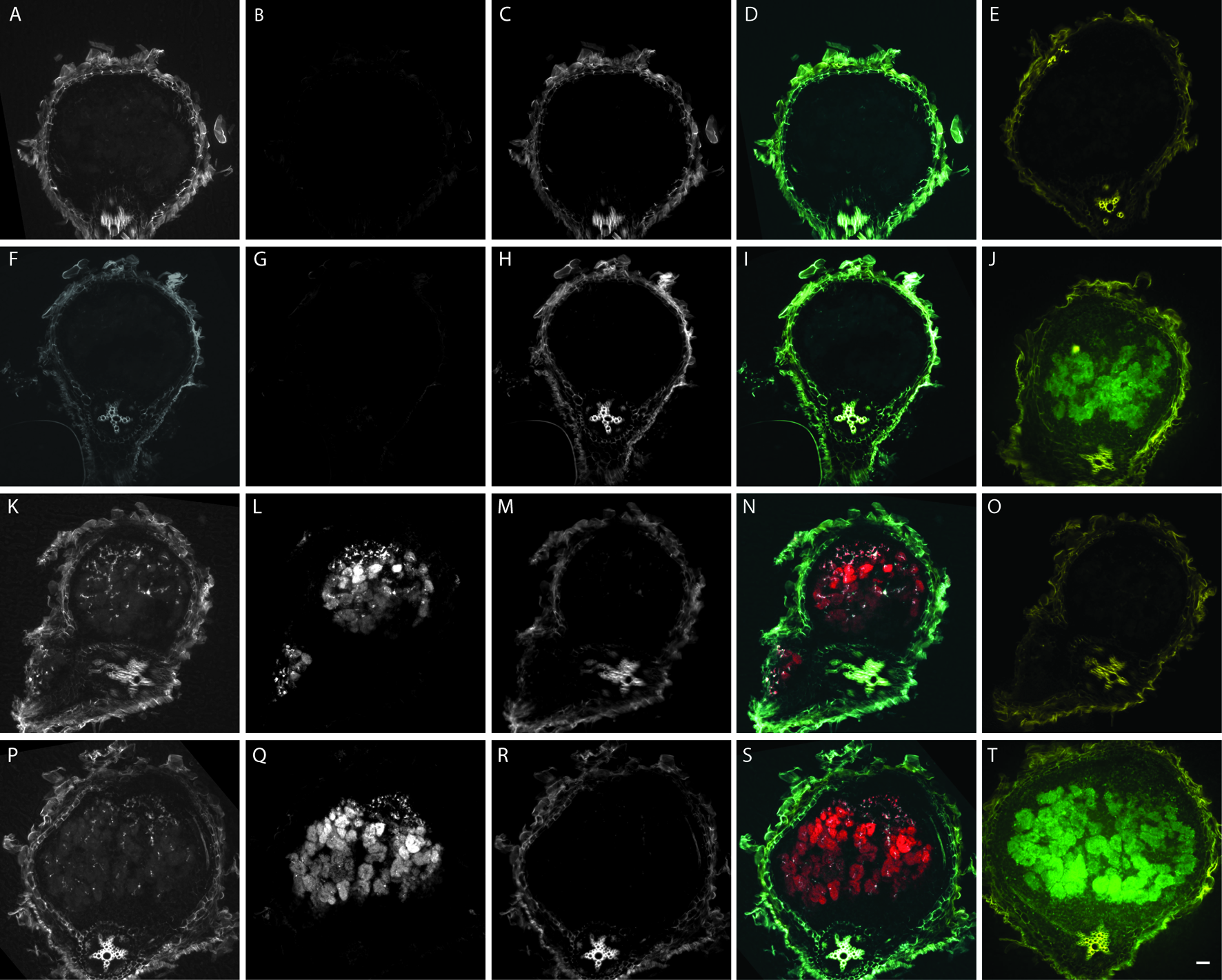

Supplement: FIGURE S1 — Fluorescence microscopy of Sinorhizobium meliloti control strains. The first row (A–E) shows nodules from plants inoculated with the reporter-less, wild type strain CL150. The second row (F–J) shows nodules inoculated with strain CL227, which contains the PnifH-uidA fusion but not the pCL301 plasmid with PexoY-mTFP and PbacA-mCherry. The third row (K–O) shows nodules inoculated with CL296, which contains the pCL301 plasmid but not the PnifH-uidA fusion. The fourth row (P–T) shows nodules from the final reporter strain CL304. The first column (A,F,K,P) shows the mTFP signal, the second column (B,G,L,Q) shows the mCherry signal, the third column (C,H,M,R) the UV signal, the fourth column (D,I,N,S) an overlay of the mTFP, mCherry, and UV signal, and the fifth column (E,J,O,T) shows an overlay of the uidA and UV signals. All nodules were harvested at 14 dpi nodules from M. truncatula A17. Scale bar: 50 μm. [file Image_1.TIF]
