# Supplementary material for: Characterization of Novel Plant Symbiosis Mutants Using a New Multiple Gene-Expression Reporter Sinorhizobium meliloti Strain
Source: Front Plant Sci. 2018 Feb 7;9:76. doi: 10.3389/fpls.2018.00076 (PMC5808326; doi:10.3389/fpls.2018.00076)
Supplement: TABLE S1 — Plasmids used in this study. [file Table_1.DOCX]

Supplementary Material

Characterization of novel plant symbiosis mutants using a new multireporter *S. meliloti* strain

Claus Lang, Lucinda S. Smith, Sharon R. Long^*^

*** Correspondence:** Sharon R. Long: SRL@stanford.edu

**Supplementary Table S1: Plasmids used in this study**

| **Plasmid** | **Description** | **Source** |
| --- | --- | --- |
| pQDNO3 | pDG1 derivative with mCherry | Haney, C.H., and Long, S.R. (2010). Plant flotillins are required for infection by nitrogen-fixing bacteria. Proc Natl Acad Sci USA 107, 478-483. |
| pNCS-mTFP1 | *mtfp* template | Allele Biotechnology |
| pVO155 | *uidA* template | Oke, V., and Long, S.R. 1999. Bacterial genes induced within the nodule during the Rhizobium-legume symbiosis. Mol Microbiol 32:837-849. |
| pCAP77 | *rhaS* insertion vector | Pinedo, C.A., and Gage, D.J. 2009. Plasmids that insert into the rhamnose utilization locus, *rha*: a versatile tool for genetic studies in *Sinorhizobium meliloti*. J Mol Microbiol Biotechnol 17:201-210. |
| pCL141 | pCAP77 + *uidA* | this study |
| pCL146 | pCAP77 + *mtfp* | this study |
| pCL169 | P*nifH-uidA* in pCAP77 | this study |
| pCL181 | P*exoY-mTFP* in pCAP77 | this study |
| pMB393 | pBBRMCS1 derivative with spectinomycin resistance | Barnett, M.J., Oke, V., and Long, S.R. (2000). New genetic tools for use in the Rhizobiaceae and other bacteria. Biotechniques 29, 240-242, 244-245. |
| pLS1 | P*exoY-mTFP* in pMB393 | this study |
| pCL301 | P*bacA-mcherry* in pLS1 | this study |
| pXLGD4 | *hemA::lacZ* | Leong, S.A., Williams, P.H., and Ditta, G.S. 1985. Analysis of the 5' regulatory region of the gene for delta-aminolevulinic acid synthetase of *Rhizobium meliloti*. Nucleic Acids Res 13:5965-5976. |
| pMB393 | pBBRMCS1 derivative with spectinomycin resistance | Barnett, M.J., Oke, V., and Long, S.R. (2000). New genetic tools for use in the Rhizobiaceae and other bacteria. Biotechniques 29, 240-242, 244-245. |
