# Supplementary material for: Characterization of Novel Plant Symbiosis Mutants Using a New Multiple Gene-Expression Reporter Sinorhizobium meliloti Strain
Source: Front Plant Sci. 2018 Feb 7;9:76. doi: 10.3389/fpls.2018.00076 (PMC5808326; doi:10.3389/fpls.2018.00076)
Supplement: TABLE S2 — Primers used in this study. fw - forward primer; rev - reverse primer; P - 5′-phosphorylated primer. [file Table_2.docx]

Supplementary Material

Characterization of novel plant symbiosis mutants using a new multireporter *S. meliloti* strain

Claus Lang, Lucinda S. Smith, Sharon R. Long^*^

*** Correspondence:** Sharon R. Long: SRL@stanford.edu

Supplementary Table S2: Primers used in this study. fw - forward primer; rev - reverse primer; P - 5'-phosphorylated primer

| **Primer** | **Description** | **Sequence** |
| --- | --- | --- |
| CL527 | mcherry fw | AGGAGATATACATACCCATGGTGAGCAAGGGCGAGGAG |
| CL528 | mcherry rev, smaI | PCCCGGGTTACTTGTACAGCTCGTCCATGCCG |
| CL523 | bacA promoter fw1 | PAGCCCGCCTAATGAGCGGGCTTTTTTTTGCTACGCTTTGCCGACCTTG |
| CL525 | bacA promoter rev1 | TCACCATGGGTATGTATATCTCCTGTTTCGGGGAGGTCAAGGA |
| CL348 | exoY upstream fw1 | PAGCTTCAGTGCAGAATGATGCGTC |
| CL349 | exoY upstream fw2 | TCAGTGCAGAATGATGCGTC |
| CL350 | exoY upstream rev1 | GTACCCGTTGGCGATTGTCGGTC |
| CL351 | exoY upstream rev2 | PCCGTTGGCGATTGTCGGTC |
| CL328 | nifH upstream fw1 | PAGCTTCACAAGGGCTTCCACGCAC |
| CL329 | nifH upstream fw2 | TCACAAGGGCTTCCACGCAC |
| CL330 | nifH upstream rev1 | GTACCTTGTTGTTTAAGCTATTTC |
| CL331 | nifH upstream rev2 | PCTTGTTGTTTAAGCTATTTC |
| CL232 | mTFP fw1 | CAGAGGATCTCGACCATGGTGAGCAAGGGCGAGGAG |
| CL233 | mTFP fw2 | GTACCAGAGGATCTCGACCATGGTGAGCAAGGGCGAGGAG |
| CL234 | mTFP rev1 | GTACTTACTTGTACAGCTCGTCCATGCCG |
| CL235 | mTFP rev2 | TTACTTGTACAGCTCGTCCATGCCG |
| CL228 | uidAfw1 | CAGAGGATCTCGACCATGGTCCGTCCTGTAGAAAC |
| CL229 | uidAfw2 | GTACCAGAGGATCTCGACCATGGTCCGTCCTGTAGAAAC |
| CL230 | uidArev1 | GTACTTATTGTTTGCCTCCCTGCTGCGGT |
| CL231 | uidArev2 | TTATTGTTTGCCTCCCTGCTGCGGT |
