# Supplementary material for: Characterization of Novel Plant Symbiosis Mutants Using a New Multiple Gene-Expression Reporter Sinorhizobium meliloti Strain
Source: Front Plant Sci. 2018 Feb 7;9:76. doi: 10.3389/fpls.2018.00076 (PMC5808326; doi:10.3389/fpls.2018.00076)
Supplement: TABLE S3 — S. meliloti strains used in this study. [file Table_3.DOCX]

Supplementary Material

Characterization of novel plant symbiosis mutants using a new multireporter *S. meliloti* strain

Claus Lang, Lucinda S. Smith, Sharon R. Long^*^

*** Correspondence:** Sharon R. Long: SRL@stanford.edu

**Supplementary Table S3: *S. meliloti* strains used in this study**

| **Strain** | **Description** | **Reference** |
| --- | --- | --- |
| CL150 | *S. meliloti* Rm1021 with corrected point mutations in *pstC* and *ecfR1* | Schlüter, J.P., Reinkensmeier, J., Barnett, M.J., Lang, C., Krol, E., Giegerich, R., Long, S.R., and Becker, A. (2013). Global mapping of transcription start sites and promoter motifs in the symbiotic alpha-proteobacterium *Sinorhizobium meliloti* 1021. BMC Genomics 14, 156. |
| CL227 | CL150 derivative with pCL141 (P*_nifH_-uidA*) genomic integration in *rhaS* locus | this study |
| CL296 | CL150 with pCL301 | this study |
| LS121 | CL227 with pXLGD4 | this study |
| CL304 | LS121 with pCL301 | this study |
